# Chikungunya: important lessons from the Jamaican experience

**DOI:** 10.26633/RPSP.2017.60

**Published:** 2017-07-03

**Authors:** Jacqueline Duncan, Kelly Ann Gordon-Johnson, Marshall K Tulloch-Reid, Colette Cunningham-Myrie, Kacey Ernst, Nathlee McMorris, Andriene Grant, Marcia Graham, Daisylyn Chin, Karen Webster-Kerr

**Affiliations:** 1 Kingston & St. Andrew Health Department Kingston & St. Andrew Health Department Kingston Jamaica Kingston & St. Andrew Health Department, Kingston, Jamaica; 2 Ministry of Health Ministry of Health Kingston Jamaica Ministry of Health, Kingston, Jamaica.; 3 Epidemiology Research Unit Caribbean Institute for Health Research Kingston Jamaica Epidemiology Research Unit, Caribbean Institute for Health Research, University of the West Indies, Kingston, Jamaica.; 4 Department of Community Health and Psychiatry University of the West Indies, Kingston, Jamaica Kingston Jamaica Department of Community Health and Psychiatry, University of the West Indies, Kingston, Jamaica.; 5 Epidemiology and Biostatistics Department Mel & Enid Zuckerman College of Public Health TusconArizona United States of America Epidemiology and Biostatistics Department, Mel & Enid Zuckerman College of Public Health, University of Arizona, Tuscon, Arizona, United States of America.; 6 National Public Health Laboratory National Public Health Laboratory Kingston Jamaica National Public Health Laboratory, Kingston, Jamaica.; 7 St. James Health Services St. James Health Services Montego Bay Jamaica St. James Health Services, Montego Bay, Jamaica.

**Keywords:** Chikungunya virus, epidemics, Jamaica, Virus chikungunya, epidemias, Jamaica

## Abstract

**Objectives.:**

To describe the clinical presentation of chikungunya virus (CHIKV) illness in adults during the 2014 outbreak in Jamaica and to determine the predictive value of the case definition.

**Methods.:**

A cross-sectional study was conducted using clinical data from suspected cases of CHIKV that were reported to the Ministry of Health in April – December 2014. In addition, charts were reviewed of all individuals over 15 years of age with suspected CHIKV based on a diagnosis of CHIKV or “acute viral illness” that presented to four major health centers in Jamaica during the week prior to and the peak week of the epidemic. Data abstracted from these charts using a modified CHIKV Case Investigation Form included demographics, clinical findings, and laboratory tests.

**Results.:**

In 2014, the Ministry of Health of Jamaica received 4 447 notifications of CHIKV infection. PCR testing was conducted on 137 suspected CHIKV cases (56 men and 81 women; median age 28 years) and was positive for 89 (65%) persons. In all, 205 health charts were identified that met the selection criteria (51 men and 154 women, median age 43 years). The most commonly reported symptoms were arthralgia (86%) and fever (76%). Of those who met the epidemiologic case definition for CHIKV as defined by the Pan American Health Organization, only 34% had this diagnosis recorded. Acute viral illness was the most frequently recorded diagnosis (n = 79; 58%).

**Conclusions.:**

Broader case definitions for acute CHIKV illness may be needed to identify suspected cases during an outbreak. Standardized data collection forms and validation of case definitions may be useful for future outbreaks.

## RESUMEN

**Objetivo.:**

Describir el cuadro clínico de la infección por el virus del chikungunya (CHIKV) en los adultos durante el brote del 2014 en Jamaica y determinar el valor predictivo de la definición de caso.

**Métodos.:**

Se realizó un estudio transversal con los datos clínicos de los presuntos casos de CHIKV que se notificaron al Ministerio de Salud entre abril y diciembre del 2014. Además, se examinaron las historias clínicas de todos los mayores de 15 años con presunto cuadro de CHIKV, es decir con diagnóstico de CHIKV o de “virosis aguda”, que acudieron a cuatro grandes centros de salud de Jamaica durante la semana de máxima incidencia de la epidemia y la semana anterior. De las historias se extrajo, mediante un formulario modificado para el estudio de casos de CHIKV, la siguiente información: datos demográficos, resultados clínicos y análisis de laboratorio.

**Resultados.:**

En el 2014, el Ministerio de Salud de Jamaica recibió 4 447 notificaciones de infección por el CHIKV. Se realizó la RCP de 137 presuntos casos de CHIKV (56 varones y 81 mujeres; mediana de edad de 28 años), que dio positivo en 89 personas (el 65%). En total, se encontraron 205 historias clínicas que reunían los criterios de selección (51 varones y 154 mujeres; mediana de edad de 43 años). Los síntomas comunicados con mayor frecuencia fueron las artralgias (86%) y la fiebre (76%). De los que se ajustaron a la definición epidemiológica de caso de CHIKV de la Organización Panamericana de la Salud, solo en el 34% se había registrado ese diagnóstico. La virosis aguda fue el diagnóstico registrado con mayor frecuencia (n = 79; 58%).

**Conclusiones.:**

Es posible que se necesiten definiciones de caso más amplias para la infección aguda por CHIKV a fin de identificar los casos sospechosos durante un brote. Los formularios estandarizados para la recopilación de datos y la validación de las definiciones quizá resulten útiles para otros brotes en el futuro.

Chikungunya is caused by the chikungunya virus (CHIKV), a ribonucleic acid virus belonging to the *Alphavirus *genus of the *Togaviridae *family (1, 2). It is a mosquito-borne virus transmitted predominantly by *A. aegypti *and *A. albopictus *and was first reported in Tanzania in 1952 (1). Subsequent CHIKV outbreaks were documented in Africa, Asia, and some parts of Europe (3–6).

In December 2013, CHIKV was first detected in the Region of the Americas, on islands in the Caribbean (7, 8). Jamaica recorded its first confirmed CHIKV case in August 2014 (9). Most recently, CHIKV has been reported in over 30 countries and territories in the Americas, having spread from the Caribbean throughout the Region and infecting more than 1 million people (10–12).

The effect of CHIKV on the Caribbean population has been unprecedented. As there was little immunity to the virus, a large proportion of the population was estimated to be infected. Jamaica, like most other Latin American and Caribbean countries, has limited local and regional facilities for analyzing specimens using polymerase chain reaction (PCR) testing—the gold standard when performed within 2 days of onset. Additionally, the CHIKV antibody testing kits had low sensitivity for identifying cases of the disease (13). At the peak of the epidemic, local and referral laboratory services were quickly overwhelmed by the number of cases that presented for care. As a result, most individuals were diagnosed based on clinical impression or use of a symptom-based case definition. Laboratory-confirmed cases, therefore, likely reflect only a fraction of the total cases that occurred in Jamaica.

This probable underreporting is of importance, especially since there are significant gaps in our knowledge of the natural history of CHIKV—in particular, the long term sequelae of the disease. Given the resource constraints and lack of laboratory confirmation of CHIKV cases, future studies that examine longterm consequences of the 2014 outbreak will have to rely on patient records that list symptoms and clinical diagnoses to identify CHIKV cases. Therefore, evaluation of this approach to case classification is critical prior to implementing broader research.

The objective of this study was to determine the predictive ability of the case definition used during 2014 chikungunya epidemic in Jamaica. The case definition was evaluated against the PCR results of cases with blood collected within 2 days of symptom onset; and in addition, the case notes from a primary care clinic in each of Jamaica’s health authorities were examined to capture the recorded symptoms and physician diagnoses, and to assess agreement between each diagnosis and the case definition.

## MATERIALS AND METHODS

### Setting and sampling

Jamaica’s health system is a privatepublic mix overseen by the Ministry of Health. The public health system is divided into four Regional Health Authorities (RHAs). Among the RHAs, primary and secondary care services are provided at 23 hospitals and more than 200 primary care health centers island-wide. These health centers vary from Type 1 clinics that provide maternal and child care to Type 4/5 clinics that offer specialized clinical services, including laboratory services. Health centers are linked to regional hospitals and refer patients to secondary or tertiary care facilities as necessary. The smaller, primary care clinics are community based, while the larger clinics (where a physician is available) draw patients from a wider geographic area.

Specialized laboratory support for government public health services is centralized at the National Public Health Laboratory, though many private facilities also provide diagnostic services. Specimens for emerging infections are usually sent to the regional laboratory based at the Caribbean Public Health Agency (CARPHA). By law, notifiable diseases, such as CHIKV, must be reported to the public health authorities.

### Ministry of health data

Reporting of notifiable diseases is done on an ongoing basis to the Ministry of Health through active and passive surveillance from medical practitioners, sentinel sites at primary health care centers, and all major hospitals. There are three classes of notifiable diseases. Class 1 notifiable diseases include all outbreaks and must be reported on suspicion to the Ministry of Health or a local health department within 24 hours of contact with the health system. CHIKV was designated a Class 1 notifiable disease. During the 2014 CHIKV outbreak, 4 447 cases were reported to the Ministry of Health. In April–December 2014, the Ministry’s Case Investigation Form for CHIKV was used to collect surveillance data for persons who met the CHIKV case definition. PCR testing was done at the CARPHA Laboratory using the Real Time PCR (RT-PCR) protocol issued by the Centers for Disease Control and Prevention (Atlanta, Georgia, United States; 14) for persons residing in a community where CHIKV transmission had not been previously established.

### Case definition

The Ministry of Health of Jamaica defined suspected and confirmed cases of CHIKV based on epidemiological criteria defined by the Pan American HealthOrganization (PAHO; 15). A suspected case was defined as fever of acute onset of > 101.3oF (38.5 oC) *and *severe arthralgia (or arthritis) not explained by other medical conditions in a person who resides in or had visited an epidemic or endemic area within 2 weeks prior to symptom onset. A confirmed case was defined as a suspected case with a positive result by any of the following CHIKV specific laboratory tests: viral isolation, detection of viral RNA by RT-PCR, detection of IgM in a single serum sample (collected during acute or convalescent phase), or 4-fold increase in CHIKVspecific antibody titers (samples collected at least 2 – 3 weeks apart).

An operational definition was used for chart reviews due to the absence of serological confirmation for most cases of CHIKV during the outbreak. Charts were reviewed for persons who met the following criteria:

- More than 15 years of age AND
- Diagnosed with CHIKV infection OR
- Diagnosed with acute viral illness.

### Chart reviews

One Type 4/5 public health center located an urban area in each RHA was intentionally selected. In May – June 2015, charts were reviewed of persons with suspected CHIKV who had visited the selected health centers in each health region. The peak week of the CHIKV outbreak was determined by reviewing the Ministry of Health’s surveillance data. The line listing of patients visiting each health center during the study period was reviewed to identify suspected cases based on the operational definition.

A modified version of the Ministry of Health’s Case Investigation Form for CHIKV (Supplementary Materials) was used to collect retrospective data on demographics, comorbidities, clinical presentation, medication use, diagnostic laboratory tests (if available), clinician diagnosis, and disposition of persons identified from the line listing.

### Statistical analysis

The CHIKV confirmation rate was calculated by dividing the number of PCR positive patients by those who met the case definition. For the chart reviews, frequencies of recorded symptoms, disaggregated by sex, age, RHA, and comorbid medical conditions were determined and the proportions were calculated. Chisquare and Fisher’s exact tests were used to investigate associations between the recorded symptoms and these characteristics. An epidemiologic definition (fever and arthritis in areas with local transmission of CHIKV in the last 15 days) was used as the gold standard definition for chart reviewed cases without laboratory diagnosis. Patients with missing or absent data on fever and arthritis did not meet the case definition. The proportion of physicians recording CHIKV as a final diagnosis according to the study’s epidemiologic case definition was determined. Agreement between the epidemiologic case definition and the physician’s diagnosis was calculated (*P* < 0.05). IBM SPSS Statistics software, version 20 (SPSS Inc., an IBM company, Chicago, Illinois, United States) was used to perform the analyses.

Ethical approval was not required as the activities were solely the public health response by the Ministry of Health to an outbreak of CHIKV in Jamaica. Personal information was handled in a confidential manner, in keeping with the Ministry of Health’s guidelines. Only aggregate data (without identifiers) was shared with any researchers who were not members of the Ministry of Health surveillance team.

## RESULTS

### Ministry of Health data

From April – December 2014, the Ministry of Health submitted blood samples from 137 suspected CHIKV cases for PCR testing. The cases were predominantly female (59%) and their age ranged from less than 1 year to 85 years of age, with a median of 28 years (Interquartile range (IQR) = 17–45 years). A total of 89 cases (65%) were confirmed as CHIKV positive by PCR testing.

### Health chart review

In all, the medical records of 205 individuals meeting the operational case definition were analyzed. Table 1 presents the frequency of reported symptoms in these patients. Of those with a physician diagnosis of “CHIKV” or “acute viral illness,” arthralgia/arthritis was the most frequently recorded symptom (86%), followed by fever (76%) and skin manifestation (34%). There were no significant differences in recorded symptoms by age, sex, medical history, or geographic area. With the exception of fever and arthritis/arthralgia, other clinical symptoms of interest were not recorded in 20% – 30% of the charts reviewed (Table 1).

**Table 1.** Frequency of recorded chikungunya symptoms, disaggregated by sex, during the week prior to and the peak week of the epidemic, Jamaica, 2014

Symptom	Sex				Total	
	Female (*n*)	%	Male (*n*)	%	n	%
Arthralgia/arthritis						
Yes	134	87.0	44	86.3	178	86.8
No	13	8.4	6	11.8	19	9.3
Not applicable/stated	7	4.5	1	2.0	8	3.9
Fever						
Yes	117	76.0	38	74.5	155	75.6
No	29	18.2	13	25.5	42	20.5
Not applicable/stated	8	5.2	0	0.0	8	3.9
Skin manifestations						
Yes	61	39.6	9	17.6	70	34.1
No	63	40.9	31	60.8	94	45.9
Not applicable/stated	30	19.5	11	21.6	41	20.0
Skin manifestations						
Yes	61	39.6	9	17.6	70	34.1
No	63	40.9	31	60.8	94	45.9
Not applicable/stated	30	19.5	11	21.6	41	20.0
Headache						
Yes	45	29.2	15	29.4	60	29.3
No	80	51.9	25	49.0	105	51.2
Not applicable/stated	29	18.8	11	21.6	40	19.5
Myalgia						
Yes	35	22.7	14	27.5	49	23.9
No	87	56.5	28	54.9	115	56.1
Not applicable/stated	32	20.8	9	17.6	41	20.0
Asthenia						
Yes	17	11.0	8	15.7	25	12.2
No	96	62.3	33	64.7	129	62.9
Not applicable/stated	41	26.6	10	19.6	51	24.9
Back pain						
Yes	17	11.0	4	7.8	21	10.2
No	98	63.6	32	62.7	130	63.4
Not applicable/stated	39	25.3	15	29.4	54	26.3
Periarticularoedema						
Yes	19	12.3	2	3.9	21	10.2
No	94	61.0	34	66.7	128	62.4
Not applicable/stated	41	26.6	15	29.4	56	27.3
Nausea						
Yes	8	5.2	4	7.8	12	5.9
No	107	69.5	36	70.6	143	69.8
Not applicable/stated	39	25.3	11	21.6	50	24.4
Vomiting						
Yes	3	1.9	2	3.9	5	2.4
No	111	72.1	38	74.5	149	75.1
Not applicable/stated	40	26.0	11	21.6	51	24.9

***Source:*** Prepared by the authors from the study data.

**Table 2.** Characteristics of cases meeting the operational case definition of chikungunya used for chart review during the week prior to and the peak week of the epidemic, Jamaica, 2014

Characteristics of participants	Frequency	
	n	%
Sex		
Female	154	75.1
Male	51	24.9
Age group		
15 – 24 years	46	22.4
25 – 59 years	129	62.9
60 years and older	30	14.6
Comorbidity Hypertension:		
Yes	69	33.7
No	110	53.7
Diabetes:		
Yes	22	10.7
No	143	69.8
Not stated	40	19.5
Pre-existing artdritis		
Yes		
No	151	73.7
Not stated	41	20.0

***Source:*** Prepared by the authors from the study data.

**FIGURE 1.**
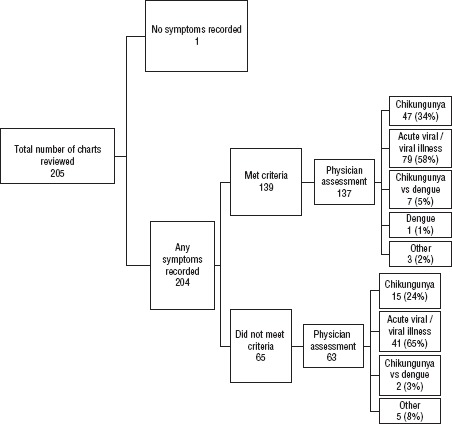
Physician diagnosis process flow according to chikungunya status based on epidemiologic diagnostic criteria defined by the Pan American Health Organization, during either the week prior to or the peak week of the chikungunya outbreak, Jamaica, 2014

***Source:*** Prepared by the authors from the study data.

The characteristics of the patients whose illness met the chart review’s operational case definition for CHIKV are presented in Table 2. The majority was female (75%), with a median of 43 years of age (IQR: 26–54 years). Hypertension (33.7%) was the most frequently reported comorbidity. For 204 (99.5%), some clinical symptoms were recorded on a health chart by the attending health care provider. Figure 1 classifies the patients according to symptoms recorded.

Of those persons with symptoms recorded, 139 patients (68%) met the PAHO epidemiologic case definition and among these, CHIKV was recorded on 47 (34%) of these health charts. Other diagnoses recorded were: “acute viral illness/viral illness” (58%); “CHIKV versus dengue” (5%); and “dengue” (1%). A total of 65 patients (32%) did not meet the PAHO epidemiologic case definition and, of these, 63 had a physician diagnosis recorded on their chart. A diagnosis of CHIKV was made for 15 of the 63 patients (24%) who did not meet the PAHO epidemiological case definition. There was poor agreement between the PAHO epidemiological case definition and physician diagnosis (Kappa = 8%).

## DISCUSSION

During the 2014 chikungunya epidemic in Jamaica, approximately twothirds of those presenting with CHIKV symptoms that met the Ministry of Health’s case definition and who were PCR-tested, were positive for the disease. The medical records at the primary care clinics showed that fever and joint pain were the most commonly recorded symptoms. Details of other symptoms were often missing, leaving two possibilities: the symptoms were either not present or not recorded. Of the health charts that met the PAHO epidemiologic case definition, only 34% had a CHIKV diagnosis recorded by a physician, acute viral illness being the more frequent diagnosis.

### Predictive value of the case definition

The predictive value of the case definition for CHIKV diagnosis may be higher than might be expected in the general population. This was because the cases selected for PCR testing met the Ministry definition for CHIKV and had also had a blood sample collected within 2 days of symptom onset. In other studies, there were variations in the case definitions, diagnostic tests, and PCR timing after symptom onset (14, 16).

To the authors’ knowledge, there is only one other published study from the Caribbean in which suspected cases that met a case definition like that of Jamaica’s had PCR, IgM, and IgG antibody testing performed using a standard protocol based on the blood sample timing (17). Of the 1 502 suspected cases evaluated in that study in St. Martin (17), 38% were confirmed CHIKV and 4% were confirmed dengue, with 16 patients co-infected by both viruses. Other studies that have evaluated laboratory tests for CHIKV diagnosis did not compare clinical data to laboratory results, and therefore, did not evaluate the validity or predictive value of the clinical case definition (13). This is important since laboratory testing is likely to be unavailable in resource-poor settings during outbreaks, and health care providers will have to rely on case definitions to identify CHIKV.

The high confirmation rate in the present study, among persons meeting the case definition, may be a result of the high prevalence of infection during the 2014 outbreak in Jamaica. We believe that this estimate may be valid using the PAHO epidemiologic definition in the chart reviews obtained at the peak of the epidemic in each of Jamaica’s health regions.

### Physician-recorded diagnosis

“Acute viral illness” was the most commonly recorded diagnosis in the primary care setting, even when patients had the two symptoms included in the PAHO CHIKV case definition, i.e., fever and joint pain. However, since a diagnosis of CHIKV should be made only in the presence of these symptoms *and *in the absence of other possible viral illnesses, some physicians may have declined to definitively label a case as CHIKV. This is important when conducting medical record reviews since using physiciandiagnosed CHIKV as inclusion criteria may exclude a large proportion of persons with the disease. At the time of the CHIKV outbreak in Jamaica, there were no other major viral outbreaks, so it is unlikely that the fever and joint pain seen among the population had another cause. Future retrospective studies that use medical record reviews may be advised to use broader syndromic definitions to increase case identification.

### Quality of records

Future studies may also be limited by the quality of the information recorded.In the present study, only one-quarter of the charts had symptoms recorded that could substantiate the attending physician’s impression. Most of the countries involved in this outbreak were overwhelmed by the disease and did not have organized systems for data collection. Future investigations of the CHIKV outbreak may be limited by the quality of existing records.

### Limitations

The lack of biological confirmation of suspected cases of CHIKV is an important limitation, particularly for those cases where data was missing for symptoms. PCR measurement was not available in Jamaica at the time of the epidemic onset, and was initially measured at the CARPHA laboratory in only a subgroup of the population that had a higher pre-test probability of the disease. Therefore, we were unable to assess how well the casedefinition excluded a CHIKV diagnosis. When antibody testing became available, the kits that were initially used had low sensitivity (18). The low sensitivity of these tests and the high cost of private antibody testing meant that most diagnoses were based on clinical impression.

During the peak of the outbreak in August – October 2014, the number of notifications for dengue fever also increased. A total of 919 suspected cases of dengue were reported in 2014, with 72 being laboratory confirmed. However, we were not able to comment on the possibility of dengue and CHIKV co-infection since diagnostic testing was not done routinely for both; but rather according to the Ministry of Health’s case definition for either dengue or CHIKV.

Furthermore, the chart review was limited to patients who presented to the public health care system, but an undetermined number of persons with the same symptoms may have accessed private health care. It is possible, however, that our study’s sample represents a subset of the population infected with more severe symptoms or complications. The poor recording of symptoms is also a limitation of this retrospective analysis.

## Conclusions

CHIKV is now endemic to the Caribbean; therefore, another epidemic of this magnitude is unlikely in the near future. Countries with low exposure to CHIKV may benefit from availability of standardized forms to more easily and uniformly record symptoms and to improve data quality.

Additional studies to validate the CHIKV case definition are needed, since it may be the only means of diagnosing the disease in resource-limited settings. Such studies may improve the sensitivity of case definitions, as well as contribute to the development of algorithms to differentiate diseases with similar presentation (e.g., dengue).

Laboratory capacity must be improved by encouraging Regional and international health partnerships. Greater capacity will permit diagnosis of emerging diseases in resource-limited settings and facilitate a swift response to outbreaks. Additionally, systems for storing biological samples that are pending laboratory testing would also aid with future outbreaks. Lastly, pooling of data on patients with serological testing results from other Caribbean countries may provide additional insight into chikungunya disease and its natural history.

## Acknowledgements.

The authors appreciate the Ministry of Health of Jamaica for its support of this study.

## Disclaimer.

Authors hold sole responsibility for the views expressed in the manuscript, which may not necessarily reflect the opinion or policy of the *RPSP/PAJPH *and/or PAHO.
